# Good's syndrome presenting with CMV pneumonitis and oesophageal candidiasis: A case report

**DOI:** 10.1002/rcr2.888

**Published:** 2021-12-01

**Authors:** Chin Tong Kwok, Yiu Cheong Yeung

**Affiliations:** ^1^ Department of Medicine and Geriatrics Princess Margaret Hospital Hong Kong China

**Keywords:** Good's syndrome, hypogammaglobulinaemia, renal cell carcinoma, thymoma

## Abstract

Good's syndrome is an acquired combined T‐ and B‐cell immunodeficiencies and patients are prone to opportunistic infections. The diagnosis is based on a characteristic immunoglobulin and lymphocyte subset profile, with clinical features of thymoma. Despite thymectomy, the immunodeficiencies persist and lifelong immunoglobulin replacement is necessary to prevent infections.

## Graphical abstract

Thymomas are often associated with paraneoplastic disorders. Patients with thymoma presenting with opportunistic infections should be screened for hypogammaglobulinaemia. We describe a case of Good's syndrome presenting with cytomegalovirus pneumonitis and oesophageal candidiasis, and an incidental diagnosis of renal cell carcinoma.

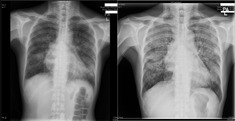

## INTRODUCTION

Thymomas are often associated with paraneoplastic disorders. Patients with thymoma presenting with opportunistic infections should be screened for hypogammaglobulinaemia. We describe a case of Good's syndrome presenting with cytomegalovirus (CMV) pneumonitis and oesophageal candidiasis, and an incidental diagnosis of renal cell carcinoma.

## CASE REPORT

A 53‐year‐old Chinese woman presented to the emergency department with a 1‐week history of fever and worsening productive cough in the context of progressive dyspnoea and unintentional weight loss over the last 6 months. She was a construction site worker and non‐smoker with a background history of depression and anxiety. Physical examination revealed normal haemodynamic status, peripheral capillary oxygen saturation (SpO_2_) of 100% (oxygen flow 3 L/min) and coarse crepitation over bilateral lower chest. Chest radiography showed bilateral lower zone consolidation. Laboratory tests revealed microcytic anaemia (haemoglobin 7.4 g/dl, mean corpuscular volume 59 fl), neutrophil‐predominant leucocytosis (white cell count 15.7 × 10^9^/L and neutrophil count 12.8 × 10^9^/L) and thrombocytosis (platelet count 439 × 10^9^/L). C‐reactive protein was elevated to 121 mg/L (reference <5 mg/L) with unremarkable procalcitonin level. Her liver and kidney function tests including globulin level were unremarkable. Microbiological workup was only positive for rhinovirus and enterovirus.

**FIGURE 1:**
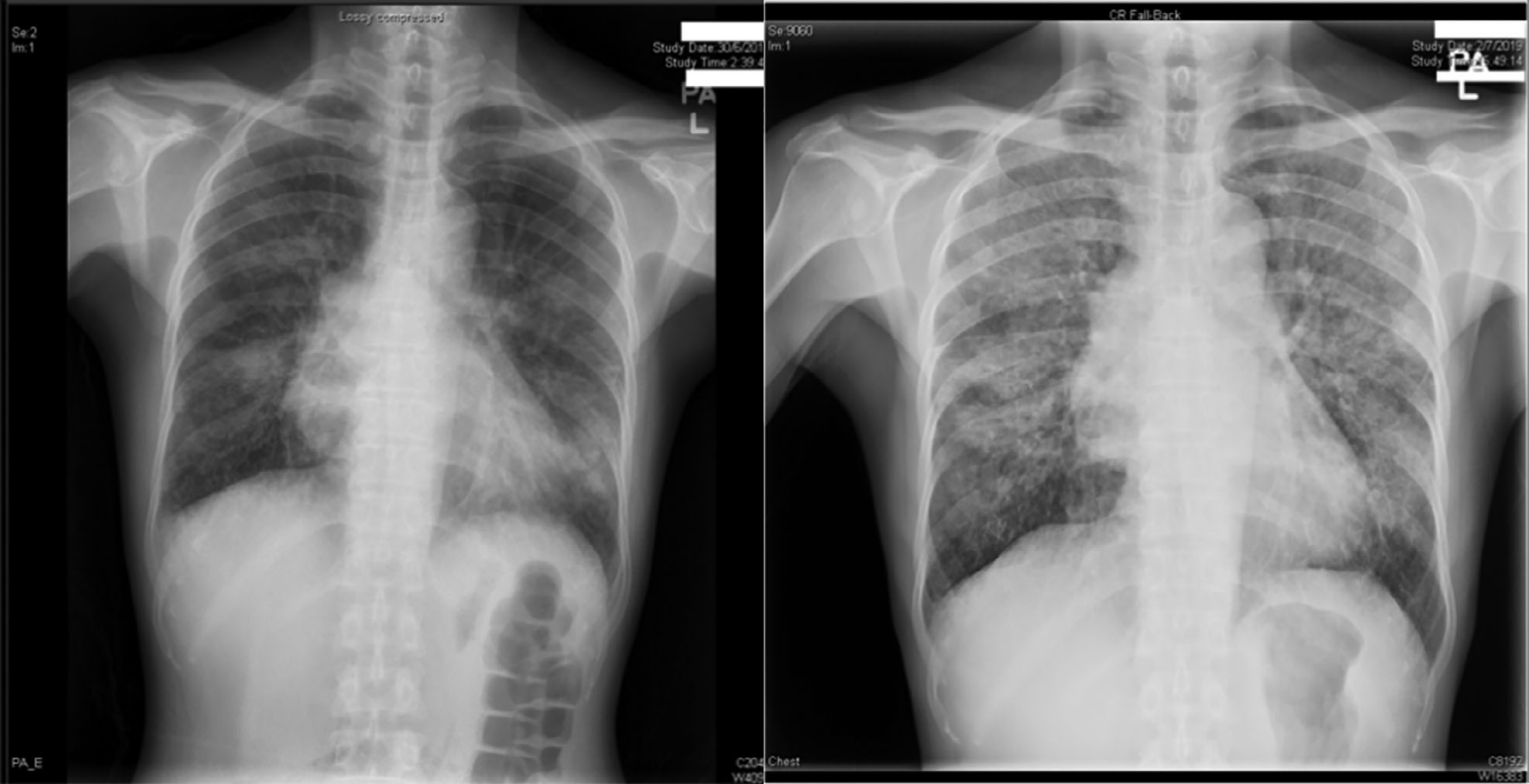
Chest x‐rays on admission (left) and on day 4 (right). Progression is evident with increase in lung infiltrate bilaterally, especially over the upper lobes. The mediastinum width was 6 cm

**FIGURE 2:**
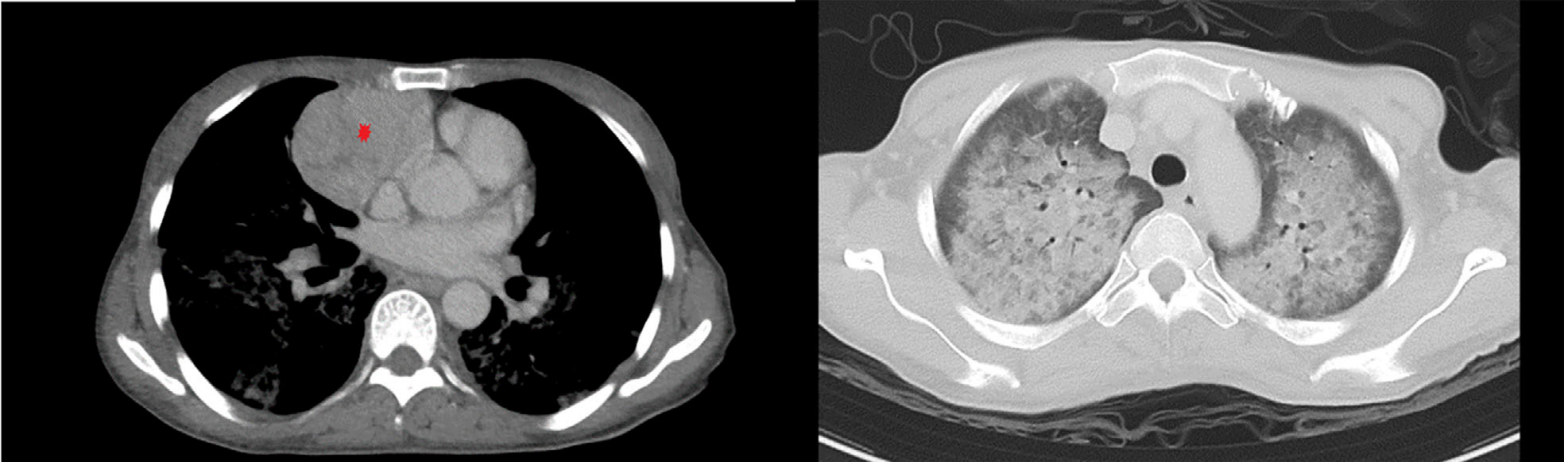
Contrast computed tomography of the thorax on day 7 of admission. Bilateral lungs confluent consolidations and ground‐glass densities with superimposed septal thickening are noted in all lobes, with upper zone predilection (right image). The anterior mediastinal soft tissue mass (marked with red star shown on the left image) measures 6.5 cm × 5.5 cm × 9.5 cm with hypo‐dense and hypo‐enhancing component. There are multiple enlarged mediastinal lymph nodes largest up to 1.2 cm at the left hilar region

Follow‐up chest x‐rays showed an increase in consolidation (Figure 1). She was initially treated with empirical intravenous amoxicillin‐clavulanic acid and oral azithromycin, and escalated to piperacillin‐tazobactam as her fever persisted. Further contrast computed tomography (CT) of the thorax demonstrated bilateral lungs confluent consolidations with ground‐glass densities and an anterior mediastinal mass (Figure 2). Bronchoscopy revealed non‐specific mucosa oedema over bilateral bronchi without endobronchial lesion bilaterally. Bronchoalveolar lavage was positive for CMV antigen and negative for bacterial, acid‐fast bacilli and fungal culture. Trans‐bronchial lung biopsy showed CMV inclusion body. The serum CMV pp65 antigen level was elevated to 10 positive cells. She was diagnosed and treated for CMV pneumonitis with ganciclovir. Ultrasound‐guided fine‐needle aspiration of the anterior mediastinal mass showed type A thymoma. Oesophagogastroduodenoscopy, as workup for her microcytic anaemia, showed diffuse yellowish white plaques along the whole oesophagus. Oesophageal biopsy showed collections of yeast‐like organisms in the horny layer eliciting a moderate inflammatory reaction consisting mainly of neutrophils. Oesophageal candidiasis was diagnosed and treated by fluconazole. Her fever subsided on day 10 without further oxygen requirement.

Investigations for immunodeficiencies revealed hypogammaglobulinaemia. Immunoglobulin A and immunoglobulin M levels were both <0.06 g/L while the immunoglobulin G level was 1.97 g/L (7–16 g/L). Lymphocyte subsets profile showed a CD19 count of 2/μl (160–708/μl), CD4 count of 409/μl (415–1418/μl), a CD8 count of 1212/μl (292–1258/μl) and a decreased CD4:CD8 ratio of 0.34 (0.62–2.7). HIV serology and autoimmune markers including anti‐nuclear antibody and rheumatoid factor were negative. Anti‐acetylcholine receptor was elevated to 0.62 nmol/L (<0.45 nmol/L).

A diagnosis of Good's syndrome was made based on the clinical features of thymoma and opportunistic infections by CMV and Candida, and the laboratory features of hypogammaglobulinaemia and CD4 T‐cell lymphopenia. She underwent thymoma excision and histopathology confirmed type AB thymoma. The patient was discharged with valganciclovir and monthly intravenous immunoglobulin replacement. Her immunoglobulin G trough level before the third dose of replacement was normalized at 11.2 g/L.

CA‐125 was elevated to 151 kIU/L (<35 kIU/L) on tumour markers screening. CT of the abdomen and pelvis identified a 2.0 × 1.9 × 1.7 cm heterogeneously enhancing exophytic lesion at the posterior aspect of the upper pole and interpolar region of the left kidney. The patient subsequently underwent laparoscopic partial nephrectomy. Histopathology confirmed renal cell carcinoma of clear cell type.

## DISCUSSION

Good's syndrome was first reported in 1954 by Robert Good as thymoma with idiopathic acquired hypogammaglobulinaemia. It is one of the paraneoplastic disorders of thymoma, apart from myasthenia gravis and pure red cell aplasia. It is characterized by a combined T‐ and B‐cell immunodeficiency including hypogammaglobulinaemia, reduced or absent B cells, CD4 T‐cell lymphopenia leading to decreased CD4:CD8 T‐cell ratio and impaired T‐cell mitogenic response.^1^ The prevalence of hypogammaglobulinaemia in patients with thymoma was reported as 2%–3%.^2^

Good's syndrome mainly affects people aged 50–60 years, with a slight female preponderance.^3^, ^4^, ^5^ Thymoma is diagnosed concurrently with hypogammaglobulinaemia in 20%–74% of cases.^3^, ^4^, ^5^ The diagnosis of thymoma preceded the emergence of hypogammaglobulinaemia or infection in 42% of patients with an interval of 3 months to 18 years in an American case series.^4^ On the contrary, thymoma was diagnosed after the documentation of hypogammaglobulinaemia or infection in 54% of patients in a Chinese case series.^3^ Therefore, thymoma and hypogammaglobulinaemia do not always occur concurrently, and would be easily missed if they were not actively looked for. Thymoma is classified by the World Health Organization based on the neoplastic epithelial cells. Type AB thymoma accounts for more than 50% of Good's syndrome cases while thymic carcinoma is only found in less than 10% of cases.^3^, ^4^, ^5^

Patients with Good's syndrome usually present with infections, diarrhoea and autoimmunity. The most common infection is sinopulmonary infection^3^, ^4^, ^5^ by *Pseudomonas aeruginosa* ^3^, ^4^ and *Haemophilus influenzae*.^4^ The common opportunistic pathogens are CMV,^3^, ^4^ *Pneumocystis jirovecii* ^3^ and Candida spp.^4^ Diarrhoea was present in up to 50% of patients, with no pathogens identified in some cases.^3^, ^4^ The most common autoimmune manifestation is pure red cell aplasia.^3^, ^4^, ^5^

Thymectomy and intravenous immunoglobulin replacement are the main treatment for Good's syndrome. Unlike myasthenia gravis which improves after thymectomy, immunodeficiency persists after thymectomy in Good's syndrome.^3^ Thus, a lifelong intravenous immunoglobulin replacement is necessary to prevent infections.^4^ Prognosis for Good's syndrome is poor due to the associated infections, haematological and autoimmune diseases,^3^, ^4^ with an estimated mortality of 46% in the observation period in a case series review.^4^ Our patient received monthly intravenous immunoglobulin and remained infection‐free 1 year after the diagnosis.

Renal cell carcinoma of clear cell type was incidentally identified in our case. A British cohort of 78 patients has previously found no association between Good's syndrome and extra‐thymic cancers including renal cell carcinoma.^5^

To avoid misdiagnosis, physicians should have a high index of suspicion of Good's syndrome when managing a patient with thymoma or mediastinal mass presenting with pneumonia or opportunistic infections. Serum immunoglobulin pattern serves as a good screening tool.

## CONFLICT OF INTEREST

Non declared.

## AUTHOR CONTRIBUTION

Chin Tong Kwok drafted the manuscript. Yiu Cheong Yeung critically revised the manuscript. Both authors have contributed substantially to the data acquisition and drafting of the manuscript, and have read and approved the final version.

## ETHICS STATEMENT

The authors declare that appropriate written informed consent was obtained for the publication of this manuscript and accompanying images.

## Data Availability

The data that support the findings of this study are available from the corresponding author upon reasonable request.
